# 1-[(1-Methyl-1*H*-imidazol-5-yl)meth­yl]-1*H*-indole-5-carbonitrile

**DOI:** 10.1107/S1600536812048404

**Published:** 2012-11-30

**Authors:** Josephus Jacobus de Jager, Vincent J. Smith

**Affiliations:** a Department of Chemistry and Polymer Science, Stellenbosch University, Private Bag X1, Matieland 7602, South Africa

## Abstract

In the title compound, C_14_H_12_N_4_, the dihedral angle between the indole ring system (r.m.s. deviation = 0.010 Å) and the imidazole ring is 77.70 (6)°. In the crystal, mol­ecules are linked by C—H⋯N hydrogen bonds. One set of hydrogen bonds forms an undulating chain running parallel to the *b*-axis direction, while the other undulating chain is parallel to the *c*-axis direction. In combination, (100) sheets result.

## Related literature

For background to farnesyl transferase, see: Chakrabarti *et al.* (2002 ▶). For the properties of related compounds, see: Bulbule *et al.* (2008 ▶), van Voorhis *et al.* (2007 ▶); de Ruyck & Wouters (2008 ▶).
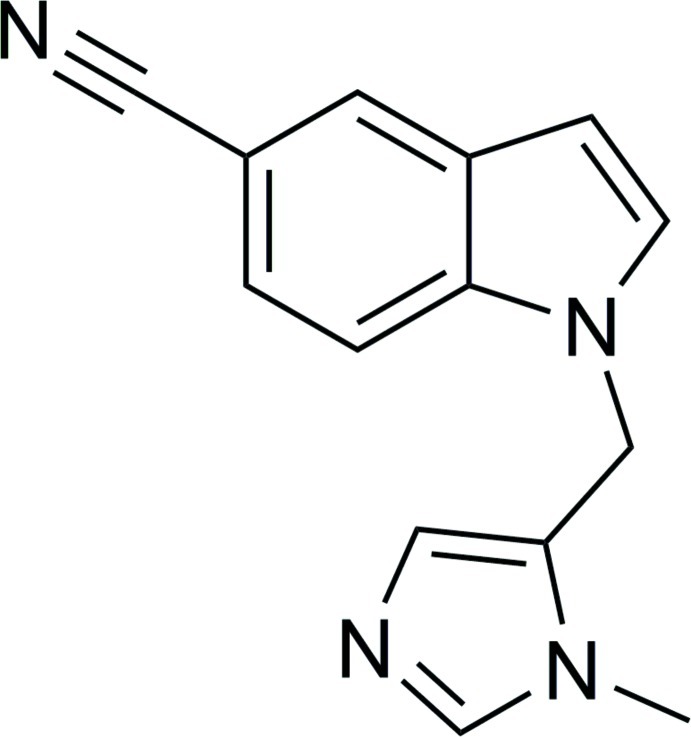


## Experimental

### 

#### Crystal data

C_14_H_12_N_4_
*M* *_r_* = 236.28
Monoclinic, 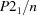

*a* = 10.9624 (16) Å
*b* = 7.8687 (12) Å
*c* = 14.292 (2) Å
β = 106.727 (2)°
*V* = 1180.6 (3) Å^3^
*Z* = 4
Mo *K*α radiation
μ = 0.08 mm^−1^
*T* = 100 K
0.10 × 0.10 × 0.02 mm

#### Data collection

Bruker APEXII CCD diffractometer
Absorption correction: multi-scan (*SADABS*; Bruker, 2009 ▶) *T*_min_ = 0.992, *T*_max_ = 0.998
36857 measured reflections
3286 independent reflections
2643 reflections with *I* > 2σ(*I*)
*R*_int_ = 0.042

#### Refinement

*R*[*F* ^2^ > 2σ(*F* ^2^)] = 0.042
*wR*(*F* ^2^) = 0.113
*S* = 1.06
3286 reflections
164 parameters
H-atom parameters constrained
Δρ_max_ = 0.32 e Å^−3^
Δρ_min_ = −0.21 e Å^−3^

### 

Data collection: *APEX2* (Bruker, 2009 ▶); cell refinement: *SAINT* (Bruker, 2009 ▶); data reduction: *SAINT*; program(s) used to solve structure: *SHELXS97* (Sheldrick, 2008 ▶); program(s) used to refine structure: *SHELXL97* (Sheldrick, 2008 ▶); molecular graphics: *X-SEED* (Barbour, 2001 ▶; Atwood *et al.*, 2003 ▶); software used to prepare material for publication: *SHELXL97*.

## Supplementary Material

Crystal structure: contains datablock(s) I, global. DOI: 10.1107/S1600536812048404/hb7000sup1.cif

Structure factors: contains datablock(s) I. DOI: 10.1107/S1600536812048404/hb7000Isup2.hkl

Supplementary material file. DOI: 10.1107/S1600536812048404/hb7000Isup3.cml

Additional supplementary materials:  crystallographic information; 3D view; checkCIF report

## Figures and Tables

**Table 1:** Hydrogen-bond geometry (Å, °)

*D*—H⋯*A*	*D*—H	H⋯*A*	*D*⋯*A*	*D*—H⋯*A*
C4—H4⋯N4^i^	0.95	2.53	3.4588 (18)	167
C13—H13⋯N4^ii^	0.95	2.57	3.4010 (18)	147

Symmetry codes: (i) 
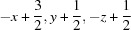
; (ii) 
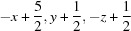
.

**Fig. 1.**
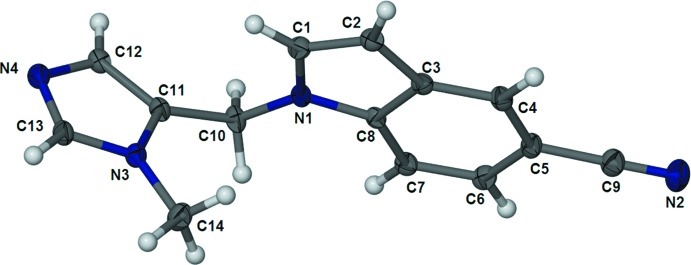
The molecular structure of the title compound with displacement ellipsoids shown at the 50% probability level.

**Fig. 2.**
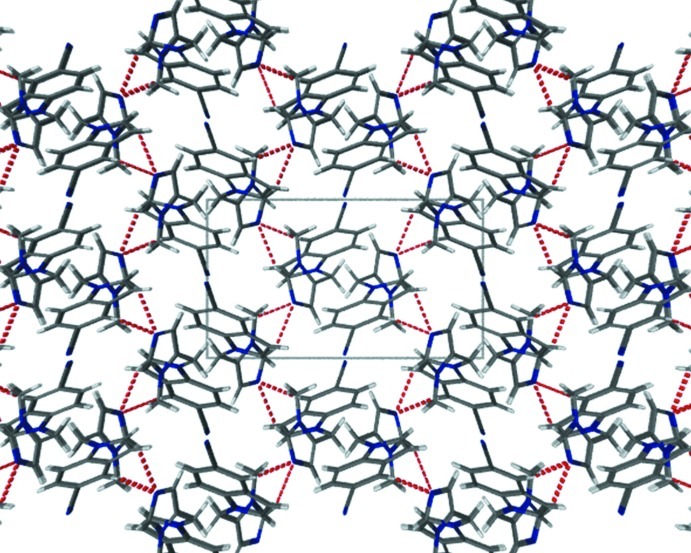
Packing diagram showing the intermolecular C—H···N hydrogen bonds.

## supplementary crystallographic information

### Comment

Protein farnesyl transferase has been identified as a drug target for its role as the sole prenylating agent in *Plasmodium falciparum* (Chakrabarti *et al.* 2002). The title compound is designed to fill some of the active site and importantly, to facilitate co-ordination to the zinc within this site *via* the imidazole side chain.

Crystalizing from a mixture of dichloromethane, toluene and n-hexane in a 18: 1: 1 ratio, crystals suitable for single-crystal X-ray diffraction were obtained. The space group was determined as *P*2_1_/*n* from the systematic absences while the unit-cell dimensions were recorded as: *a* = 10.9624 (16), *b* = 7.8687 (12), *c* = 14.2915 (21) and β = 106.727 (2). In the crystal packing the molecules associate *via* intermolecular C—H···N hydrogen bonds that form undulating chains parallel to the axial directions *b* and *c*.

### Experimental

The structure of the title compound was synthesized by the nucleophillic addition of 1*H*-indole-5-carbonitrile (195 mg, 1.37 mmol), through the use of NaH (78.6 mg, 3.28 mmol), to the hydrochloric acid salt of 5-(chloromethyl)-1-methyl-1*H*-imidazole (179 mg, 1.37 mmol) in anhydrous dimethyl formamide (5 ml). The reaction was left to proceeded for 18 h at 0 °C. The solvent was then removed *in vacuo*, after which the crude material was partitioned between water and ethyl acetate. Purification by column chromatography (1% methanol, 1% Et_3_N, 98% dichloromethane) afforded the product. Recrystallization from 90% dichloromethane, 5% n-hexane and 5% toluene produced pale yellow, block crystals.

^1^H NMR (300 MHz, CDCl_3_) δ 7.98 - 7.97 (m, 1H, ArH), 7.48 - 7.43 (m, 3H, ArH), 7.17 (s, 1H, ArH), 7.10 (d, J = 3.3 Hz, 1H, ArH), 5.30 (s, 2H), 3.34 (s, 3H, CH_3_).

### Refinement

H atoms were placed geometrically [C—H = 0.95 - 0.98 Å; with *U*_iso_(H) = 1.2 - 1.5 *U*_eq_(C)] and constrained to ride on their parent atoms.

### Crystal data

**Table:**

C_14_H_12_N_4_	*F*(000) = 496
*M**_r_* = 236.28	*D*_x_ = 1.329 Mg m^−^^3^
Monoclinic, *P*2_1_/*n*	Mo *K*α radiation, λ = 0.71073 Å
Hall symbol: -P 2yn	Cell parameters from 7965 reflections
*a* = 10.9624 (16) Å	θ = 3.0–28.6°
*b* = 7.8687 (12) Å	µ = 0.08 mm^−^^1^
*c* = 14.292 (2) Å	*T* = 100 K
β = 106.727 (2)°	Plate, colourless
*V* = 1180.6 (3) Å^3^	0.10 × 0.10 × 0.02 mm
*Z* = 4	

### Data collection

**Table:**

Bruker APEXII CCD diffractometer	3286 independent reflections
Radiation source: fine-focus sealed tube, Bruker Apex2	2643 reflections with *I* > 2σ(*I*)
Graphite monochromator	*R*_int_ = 0.042
φ and ω scans	θ_max_ = 29.8°, θ_min_ = 2.1°
Absorption correction: multi-scan (*SADABS*; Bruker, 2009)	*h* = −15→15
*T*_min_ = 0.992, *T*_max_ = 0.998	*k* = −10→10
36857 measured reflections	*l* = −19→19

### Refinement

**Table:**

Refinement on *F*^2^	Primary atom site location: structure-invariant direct methods
Least-squares matrix: full	Secondary atom site location: difference Fourier map
*R*[*F*^2^ > 2σ(*F*^2^)] = 0.042	Hydrogen site location: inferred from neighbouring sites
*wR*(*F*^2^) = 0.113	H-atom parameters constrained
*S* = 1.06	*w* = 1/[σ^2^(*F*_o_^2^) + (0.0451*P*)^2^ + 0.613*P*] where *P* = (*F*_o_^2^ + 2*F*_c_^2^)/3
3286 reflections	(Δ/σ)_max_ < 0.001
164 parameters	Δρ_max_ = 0.32 e Å^−^^3^
0 restraints	Δρ_min_ = −0.21 e Å^−^^3^

### Special details

**Table:**

Geometry. All e.s.d.'s (except the e.s.d. in the dihedral angle between two l.s. planes) are estimated using the full covariance matrix. The cell e.s.d.'s are taken into account individually in the estimation of e.s.d.'s in distances, angles and torsion angles; correlations between e.s.d.'s in cell parameters are only used when they are defined by crystal symmetry. An approximate (isotropic) treatment of cell e.s.d.'s is used for estimating e.s.d.'s involving l.s. planes.
Refinement. Refinement of *F*^2^ against ALL reflections. The weighted *R*-factor *wR* and goodness of fit *S* are based on *F*^2^, conventional *R*-factors *R* are based on *F*, with *F* set to zero for negative *F*^2^. The threshold expression of *F*^2^ > σ(*F*^2^) is used only for calculating *R*-factors(gt) *etc*. and is not relevant to the choice of reflections for refinement. *R*-factors based on *F*^2^ are statistically about twice as large as those based on *F*, and *R*- factors based on ALL data will be even larger.

### Fractional atomic coordinates and isotropic or equivalent isotropic displacement parameters (Å^2^)

**Table:**

	*x*	*y*	*z*	*U*_iso_*/*U*_eq_	
N1	0.74899 (9)	0.05550 (13)	0.10401 (7)	0.0182 (2)	
C8	0.63632 (11)	0.14156 (15)	0.06741 (8)	0.0172 (2)	
C3	0.57779 (11)	0.15677 (15)	0.14387 (8)	0.0182 (2)	
N3	1.05057 (10)	0.09402 (14)	0.16447 (8)	0.0204 (2)	
N4	1.15865 (10)	−0.14899 (14)	0.18381 (8)	0.0218 (2)	
C6	0.46523 (12)	0.28892 (17)	−0.04359 (9)	0.0222 (3)	
H6	0.4248	0.3338	−0.1066	0.027*	
C9	0.28834 (12)	0.40021 (18)	0.01344 (10)	0.0253 (3)	
C13	1.16216 (12)	0.01364 (17)	0.20734 (9)	0.0213 (3)	
H13	1.2346	0.0681	0.2495	0.026*	
C2	0.66051 (12)	0.07444 (16)	0.22788 (9)	0.0202 (2)	
H2	0.6472	0.0638	0.2904	0.024*	
N2	0.19352 (11)	0.47248 (17)	−0.00085 (10)	0.0327 (3)	
C4	0.46133 (12)	0.24195 (16)	0.12543 (9)	0.0206 (3)	
H4	0.4203	0.2545	0.1752	0.025*	
C1	0.76221 (12)	0.01428 (16)	0.20058 (9)	0.0201 (2)	
H1	0.8318	−0.0469	0.2419	0.024*	
C12	1.03687 (12)	−0.17490 (17)	0.12259 (9)	0.0209 (3)	
H12	1.0050	−0.2807	0.0937	0.025*	
C7	0.58051 (11)	0.20564 (16)	−0.02668 (9)	0.0201 (2)	
H7	0.6205	0.1923	−0.0770	0.024*	
C5	0.40688 (11)	0.30803 (16)	0.03208 (9)	0.0213 (3)	
C10	0.83783 (11)	0.01570 (18)	0.04742 (9)	0.0214 (3)	
H10A	0.8426	0.1144	0.0056	0.026*	
H10B	0.8041	−0.0819	0.0039	0.026*	
C11	0.96882 (11)	−0.02628 (16)	0.10955 (9)	0.0195 (2)	
C14	1.02309 (13)	0.27356 (18)	0.17436 (11)	0.0296 (3)	
H14A	1.0997	0.3305	0.2147	0.044*	
H14C	0.9546	0.2842	0.2054	0.044*	
H14B	0.9965	0.3265	0.1096	0.044*	

### Atomic displacement parameters (Å^2^)

**Table:**

	*U*^11^	*U*^22^	*U*^33^	*U*^12^	*U*^13^	*U*^23^
N1	0.0142 (5)	0.0216 (5)	0.0177 (5)	0.0003 (4)	0.0028 (4)	0.0016 (4)
C8	0.0135 (5)	0.0176 (5)	0.0192 (5)	−0.0028 (4)	0.0028 (4)	−0.0013 (4)
C3	0.0169 (5)	0.0178 (5)	0.0189 (5)	−0.0030 (4)	0.0035 (4)	−0.0017 (4)
N3	0.0158 (5)	0.0211 (5)	0.0230 (5)	0.0004 (4)	0.0033 (4)	0.0003 (4)
N4	0.0183 (5)	0.0244 (5)	0.0221 (5)	0.0015 (4)	0.0048 (4)	0.0025 (4)
C6	0.0197 (6)	0.0224 (6)	0.0199 (6)	−0.0009 (5)	−0.0014 (4)	0.0008 (5)
C9	0.0199 (6)	0.0251 (6)	0.0276 (6)	−0.0005 (5)	0.0013 (5)	−0.0026 (5)
C13	0.0152 (5)	0.0254 (6)	0.0222 (6)	0.0007 (5)	0.0037 (4)	0.0019 (5)
C2	0.0197 (6)	0.0218 (6)	0.0182 (5)	−0.0017 (5)	0.0042 (4)	0.0006 (4)
N2	0.0222 (6)	0.0324 (7)	0.0392 (7)	0.0041 (5)	0.0019 (5)	−0.0023 (5)
C4	0.0171 (5)	0.0215 (6)	0.0222 (6)	−0.0026 (5)	0.0043 (4)	−0.0040 (5)
C1	0.0188 (6)	0.0215 (6)	0.0183 (5)	−0.0001 (5)	0.0026 (4)	0.0024 (4)
C12	0.0188 (6)	0.0235 (6)	0.0207 (6)	−0.0010 (5)	0.0063 (4)	−0.0009 (5)
C7	0.0181 (6)	0.0224 (6)	0.0187 (5)	−0.0018 (5)	0.0033 (4)	−0.0001 (5)
C5	0.0150 (5)	0.0202 (6)	0.0256 (6)	−0.0004 (5)	0.0008 (4)	−0.0025 (5)
C10	0.0148 (5)	0.0305 (7)	0.0184 (5)	0.0006 (5)	0.0041 (4)	0.0000 (5)
C11	0.0152 (5)	0.0244 (6)	0.0185 (5)	−0.0015 (5)	0.0043 (4)	−0.0003 (4)
C14	0.0233 (6)	0.0215 (6)	0.0402 (8)	0.0016 (5)	0.0031 (6)	−0.0026 (6)

### Geometric parameters (Å, º)

**Table:**

N1—C8	1.3733 (15)	C9—C5	1.4445 (18)
N1—C1	1.3842 (15)	C13—H13	0.9500
N1—C10	1.4676 (15)	C2—C1	1.3673 (17)
C8—C7	1.4011 (16)	C2—H2	0.9500
C8—C3	1.4226 (16)	C4—C5	1.3958 (18)
C3—C4	1.3982 (17)	C4—H4	0.9500
C3—C2	1.4341 (17)	C1—H1	0.9500
N3—C13	1.3567 (16)	C12—C11	1.3707 (18)
N3—C11	1.3822 (16)	C12—H12	0.9500
N3—C14	1.4598 (17)	C7—H7	0.9500
N4—C13	1.3209 (17)	C10—C11	1.4918 (17)
N4—C12	1.3845 (16)	C10—H10A	0.9900
C6—C7	1.3817 (17)	C10—H10B	0.9900
C6—C5	1.4140 (18)	C14—H14A	0.9800
C6—H6	0.9500	C14—H14C	0.9800
C9—N2	1.1504 (18)	C14—H14B	0.9800
			
C8—N1—C1	108.65 (10)	C2—C1—H1	125.0
C8—N1—C10	124.20 (10)	N1—C1—H1	125.0
C1—N1—C10	127.14 (10)	C11—C12—N4	110.39 (11)
N1—C8—C7	129.75 (11)	C11—C12—H12	124.8
N1—C8—C3	107.70 (10)	N4—C12—H12	124.8
C7—C8—C3	122.55 (11)	C6—C7—C8	117.60 (11)
C4—C3—C8	119.00 (11)	C6—C7—H7	121.2
C4—C3—C2	134.22 (11)	C8—C7—H7	121.2
C8—C3—C2	106.78 (10)	C4—C5—C6	121.89 (11)
C13—N3—C11	106.87 (11)	C4—C5—C9	118.55 (12)
C13—N3—C14	126.29 (11)	C6—C5—C9	119.56 (12)
C11—N3—C14	126.84 (11)	N1—C10—C11	113.39 (10)
C13—N4—C12	104.82 (11)	N1—C10—H10A	108.9
C7—C6—C5	120.60 (11)	C11—C10—H10A	108.9
C7—C6—H6	119.7	N1—C10—H10B	108.9
C5—C6—H6	119.7	C11—C10—H10B	108.9
N2—C9—C5	179.32 (16)	H10A—C10—H10B	107.7
N4—C13—N3	112.35 (11)	C12—C11—N3	105.58 (11)
N4—C13—H13	123.8	C12—C11—C10	131.51 (12)
N3—C13—H13	123.8	N3—C11—C10	122.82 (11)
C1—C2—C3	106.77 (11)	N3—C14—H14A	109.5
C1—C2—H2	126.6	N3—C14—H14C	109.5
C3—C2—H2	126.6	H14A—C14—H14C	109.5
C5—C4—C3	118.35 (11)	N3—C14—H14B	109.5
C5—C4—H4	120.8	H14A—C14—H14B	109.5
C3—C4—H4	120.8	H14C—C14—H14B	109.5
C2—C1—N1	110.09 (11)		
			
C1—N1—C8—C7	−178.49 (12)	C13—N4—C12—C11	0.44 (14)
C10—N1—C8—C7	1.3 (2)	C5—C6—C7—C8	0.08 (18)
C1—N1—C8—C3	0.78 (13)	N1—C8—C7—C6	−179.67 (12)
C10—N1—C8—C3	−179.46 (11)	C3—C8—C7—C6	1.15 (18)
N1—C8—C3—C4	179.29 (11)	C3—C4—C5—C6	0.89 (18)
C7—C8—C3—C4	−1.37 (18)	C3—C4—C5—C9	−178.31 (11)
N1—C8—C3—C2	−0.42 (13)	C7—C6—C5—C4	−1.11 (19)
C7—C8—C3—C2	178.91 (11)	C7—C6—C5—C9	178.07 (12)
C12—N4—C13—N3	−0.46 (14)	C8—N1—C10—C11	161.26 (11)
C11—N3—C13—N4	0.31 (14)	C1—N1—C10—C11	−19.02 (18)
C14—N3—C13—N4	−179.36 (12)	N4—C12—C11—N3	−0.26 (14)
C4—C3—C2—C1	−179.75 (14)	N4—C12—C11—C10	176.39 (12)
C8—C3—C2—C1	−0.09 (14)	C13—N3—C11—C12	−0.02 (13)
C8—C3—C4—C5	0.32 (17)	C14—N3—C11—C12	179.65 (12)
C2—C3—C4—C5	179.95 (13)	C13—N3—C11—C10	−177.03 (11)
C3—C2—C1—N1	0.58 (14)	C14—N3—C11—C10	2.63 (19)
C8—N1—C1—C2	−0.86 (14)	N1—C10—C11—C12	112.73 (15)
C10—N1—C1—C2	179.39 (11)	N1—C10—C11—N3	−71.12 (15)

### Hydrogen-bond geometry (Å, º)

**Table:**

*D*—H···*A*	*D*—H	H···*A*	*D*···*A*	*D*—H···*A*
C4—H4···N4^i^	0.95	2.53	3.4588 (18)	167
C13—H13···N4^ii^	0.95	2.57	3.4010 (18)	147

Symmetry codes: (i) −*x*+3/2, *y*+1/2, −*z*+1/2; (ii) −*x*+5/2, *y*+1/2, −*z*+1/2.
